# Curcumin modulates gut microbiota and improves renal function in rats with uric acid nephropathy

**DOI:** 10.1080/0886022X.2021.1944875

**Published:** 2021-06-30

**Authors:** Xueling Xu, Huifang Wang, Dandan Guo, Xiaofei Man, Jun Liu, Junying Li, Congjuan Luo, Ming Zhang, Li Zhen, Xuemei Liu

**Affiliations:** Department of Nephrology, The Affiliated Hospital of Qingdao University, Qingdao, China

**Keywords:** Curcumin, uric acid nephropathy, intestinal barrier, gut microbiota

## Abstract

It is well known that the progression of hyperuricemia disease often contributes to renal dysfunction. However, there have been few studies on uric acid nephropathy (UAN), especially its relationship with gut microbiota. UAN is usually accompanied by disordered intestinal flora, and damaged gut barrier, which are closely related to tubulointerstitial fibrosis, and systemic inflammation. In previous studies, it has been confirmed that curcumin could alleviate tubulointerstitial fibrosis, and improve renal function through its antioxidant, anti-apoptotic, and anti-inflammatory efficacies. However, the effects curcumin exerts on intestinal flora in uric acid nephropathy are still unknown. Therefore, we used next-generation sequencing technology to investigate the effects of curcumin on gut microbiota in a rat model of UAN induced by adenine and potassium oxonate, and rats were randomly divided into control, model or curcumin treatment groups. The results demonstrated that, compared to the model group, the treatment group showed decreased serum uric acid (156.80 ± 11.90 μmol/L *vs.* 325.60 ± 18.65 μmol/L, *p* < 0.001), serum creatinine (66.20 ± 11.88 μmol/L *vs.* 182.20 ± 8.87 μmol/L, *p* < 0.001) and BUN level (13.33 ± 3.16 mmol/L *vs.* 36.04 ± 6.60 mmol/L, *p* < 0.001). The treatment group also displayed attenuated renal pathological lesions and metabolic endotoxemia (25.60 ± 5.90 ng/mL *vs*. 38.40 ± 4.98 ng/mL, *p* < 0.01), and improved tightly linked proteins expression. Besides, curcumin altered the gut microbiota structure in UAN rats. More specifically, curcumin treatment protected against the overgrowth of opportunistic pathogens in UAN, including *Escherichia-Shigella* and *Bacteroides*, and increased the relative abundance of bacteria producing short‐chain fatty acids (SCFAs), such as *Lactobacillus* and *Ruminococcaceae*. These results suggest that curcumin could modulate gut microbiota, fortify the intestinal barrier, attenuate metabolic endotoxemia, and consequently protect the renal function in UAN rats.

## 1. Introduction

Uric acid is the end product of purine or nucleotide metabolism, of which 2/3 is excreted by glomerular filtration and 1/3 is through intestine [1]. Hyperuricemia is one of the most common metabolic diseases worldwide, caused by increased production or inadequate renal excretion of uric acid [2]. Hyperuricemia is closely associated with the development of kidney disease. Fundamental studies have demonstrated that long-term hyperuricemia could induce deposition of urate crystals in tubulointerstitial, glomerulosclerosis, tubular damage, interstitial fibrosis, and intrarenal inflammation, leading to uric acid nephropathy (UAN) eventually [1,3,4]. Over the past few decades, both serum uric acid and the prevalence of hyperuricemia have been rapidly escalating worldwide [5–7]. A dramatic increase in the prevalence of UAN ensues, which has resulted in immense health and economic burdens. Therefore, the development of effective strategies to prevent the progression of UAN and improve renal function is critical to reducing the disease burden.

In recent years, interactions between the microbiota and their hosts have become the focus of attention, and this complex interaction is necessary for normal physiological activity. However, intestinal flora imbalance can lead to a multitude of ailments, such as diabetes, obesity, hypertensive heart disease, chronic kidney disease, inflammatory bowel disease, and some cancers [8–11]. The theory of the gut-kidney axis accounts well for bidirectional communication between the intestinal microflora and kidney disease. On one hand, impaired kidney function may result in gut flora dysbiosis [12], and on the other hand, alteration of the intestinal microflora allows the translocation of endotoxins and bacterial metabolites from the gut into the systemic circulation by disrupting the intestinal mucosal barrier, induces systemic inflammation, thus accelerating renal damage [13,14]. Hence, the intestinal flora is closely associated with the occurrence and development of kidney disease, and the regulation of gut microbiota may provide a promising strategy for the prevention and treatment of kidney disease. Interestingly, natural products as curcumin might be the novel treatment with proven safety profiles [15].

Curcumin is a natural phenolic compound derived from the rhizome of the plant curcuma longa, and it is the main ingredient of turmeric [16]. A large number of studies have confirmed that curcumin exerts a wide range of biological effects, including anti-tumor, anti-inflammatory, anti-oxidation, and anti-fibrosis [17–19]. Based on the above pharmacological effects, there have been many animal experiments and cellular experiments using curcumin to prevent and treat various kidney diseases in recent years, and even a few preliminary reports on the clinical application of curcumin in the treatment of renal diseases [20,21]. Moreover, curcumin has also been shown to have the effects of regulating intestinal flora and improving gut barrier function in multiple diseases, like diabetes, ulcerative colitis, and colorectal cancer [22–24]. Nevertheless, the precise mechanisms how curcumin improves renal function have not been completely elucidated so far, and there is no relevant research on the relationship between curcumin and intestinal microecology in kidney diseases. Therefore, the present study was performed to explore the renoprotective effect of curcumin in UAN rats and its possible mechanisms based on the theory of the gut-kidney axis.

## 2. Materials and methods

### 2.1. Animal experiments

Healthy male Wistar rats (*n* = 30, weight: 200 ± 20 g) obtained from Jinan Pengyue Experimental Animal Breeding Co., Ltd. (SCXK Lu 20190003; Jinan, China). The rats were housed under standard conditions with controlled 12 h light/dark cycles, temperature, and humidity, and had unrestrained activity and free access to water and food. After 1 week of acclimation on a normal diet, rats were randomly divided into three groups (*n* = 10 per group): control group, model group, and curcumin treatment group. The rat model of uric acid nephropathy was made by oral gavage of adenine (150 mg/kg) and potassium oxonate (250 mg/kg) for 4 weeks. After the modeling, the rats in the treatment group were intragastrically administered 200 mg/kg body weight curcumin daily for 8 weeks, whereas the other two groups received the vehicle. Body weight and body weight gain were evaluated weekly. At the end of treatment, the animals were sacrificed under diethyl ether anesthesia. The kidney, intestine, stool samples as well as blood were harvested for analysis. The experiment was carried out in strict accordance with the guidelines and ethical guidelines for experimental animals, and was approved by the Ethics Committee of Animal Experiments of the Affiliated Hospital of Qingdao University.

### 2.2. Chemical reagents

Curcumin, adenine, and potassium oxonate were purchased from Beijing Solarbio Science & Technology Co., Ltd. (Beijing, China). Antibody against ZO-1, occluding, and claudin-1 were purchased from Abcam (Cambridge, MA, USA).

### 2.3. Biochemical detection

Serum uric acid, serum creatinine, and blood urea nitrogen (BUN) were determined by automatic biochemical analyzer in the Department of Laboratory at the Affiliated Hospital of Qingdao University. Serum lipopolysaccharide (LPS) levels were measured using the endotoxin detection kit following the manufacturer’s instructions.

### 2.4. Histological analysis

After rats were sacrificed, the kidney tissues of each rat were collected. The left kidneys were accurately weighed on an electronic balance, and the left kidney weight index was calculated (the kidney coefficient = kidney mass/body weight × 100%). The right kidney was fixed in 4% buffered paraformaldehyde for 24 h, dehydrated by automatic dehydrator for 16 h, and then routinely embedded in a paraffin embedding machine for preparation of kidney tissue sections. Kidney tissue sections were then subjected to hematoxylin and eosin (HE) staining and Masson staining.

About 5 cm of ileum tissues, approximately 10 cm from the ileocecal valve, were extracted and fixed immediately in 4% paraformaldehyde, then dehydrated and embedded in paraffin wax, cut into 4 μm-thick sections and stained with hematoxylin and eosin (HE). The histopathological changes were evaluated under a light microscope, independently by two pathologists blinded to the present study design. The inflammatory damage score was the sum of extent of inflammatory infiltrate, depth of lesions, destruction of crypt, and width of lesions [25]. 10 high-power fields (×400) were randomly selected for scoring in each group, and the average value was used as the histological damage score (the possible score from 0 to 14). Scoring criteria are presented in Table 1.

**Table 1.** Histological scoring criteria.

Inflammation	Depth of lesions	Destruction of crypt	Width of lesions (%)	Score
None	None	None	0	0
Mild	Submucosa	1/3 basal crypt	1 ∼ 25	1
Moderate	Muscularis	2/3 basal crypt	26 ∼ 50	2
Severe	Serosa	Intact epithelium only	51 ∼ 75	3
**–**	**–**	Total crypt and epithelium	76 ∼ 100	4

### 2.5. Western blot analysis

The harvested ileal tissues were homogenized in RIPA lysis buffer containing PMSF and phosphatase inhibitors cocktail and centrifuged at 12,000 rpm for 20 min at 4 °C, the supernatants were collected. Protein concentrations were determined by BCA protein assay kit. Equal amounts of protein samples (20 μg) were electrophoretically separated by SDS PAGE gels and then transferred onto a PVDF membrane. The membranes were blocked with 5% powdered nonfat milk for 1 h at room temperature and were then incubated with primary antibodies against ZO-1(1:2,000), occludin (1:1,000), claudin-1 (1:1,000), or GAPDH (1:3,000) and incubated overnight at 4 °C with gentle shaking. The membranes were washed three times with TBST the following day and then incubated with a horseradish peroxidase-linked secondary antibody (1:5,000) at room temperature for 1 h. Development was performed using an enhanced chemiluminescence (ECL) system and the resultant photos were then scanned for protein level quantification using Image software. GAPDH served as the internal reference, and the relative gray values were calculated with the following formula: the relative protein expression = gray value of target protein/gray value of internal control (GAPDH).

### 2.6. Fecal samples collection and DNA extraction

A disposable sterile medical pad was placed on the operating table and the rat was held on the rat frame. The lower left side of the abdomen was stroked gently to promote defecation. Fresh feces were collected and placed into EP tubes with sterile tweezers, and then put into liquid nitrogen quickly. Thereafter, fecal samples were stored at −80 °C until assayed. The PowerSoil® DNA Isolation kit (Mo Bio Laboratories, Carlsbad, CA, USA) was used to extract DNA from the fecal samples following the manufacturer’s instructions. The concentration and purity of the DNA was measured by agarose gel electrophoresis.

### 2.7. Library construction and sequencing

After extracting the total DNA of the sample, the V3–V4 region of the bacterial 16S rRNA gene was amplified with primers 338 °F (5′-ACTCCTACGGGAGGCAGCA-3′) and 806 R (5′-GGACTACHVGGGTWTCTAAT-3′). The target sequences were amplified by PCR and its products were purified, quantified and homogenized to get a sequencing library. Then library QC was performed for constructing libraries, qualified libraries were sequenced on Illumina HiSeq 2500 (Beijing Biomarker technology). The original image data files obtained by high-throughput sequencing were converted into Sequenced Reads by Base Calling analysis, the results were stored in FASTQ (referred to as fq) format file, which contains sequence information of reads and their corresponding sequencing quality information.

### 2.8. Bioinformatic analysis

PE reads were spliced through the overlap by using FLASH software (version 1.2.11), the obtained merged sequences are original tags data (Raw Tags). To get high quality clean tags, quality filtering on the raw tags was performed by using Trimmomatic software (version 0.33). After filtering, the chimera sequences were identified and removed by using UCHIME software (version 8.1) and then the effective tags finally obtained. All sequences were clustered into operational taxonomic units (OTUs) based on 97% similarity level with USEARCH software [26] (version 10.0).

The alpha diversity index of samples based on the OTUs was evaluated by Mothur software (version 1.30.1), including Chao, Ace, Shannon and Simpson indices. Beta diversity on both weighted and unweighted unifrac were calculated to evaluate differences among groups in terms of species complexity by QIIME software (Version 1.7.0). To get the corresponding species classification information of each OTU, the OTU representative sequences can be aligned to microbial reference database, then the community composition of each sample was counted at each level (phylum, class, order, family, genus, and species). QIIME software was used to generate species richness table at different taxonomic levels, then using R language tool to draw community structure graph of samples at different taxonomic levels. LEfSe [27] (Line Discriminant Analysis (LDA) Effect Size) was used to find biomarkers with statistical difference between different groups. The LDA score threshold of the linear discriminant was set to 4.

### 2.9. Statistical analysis

Statistical analysis was performed using GraphPad Prism 6.0 (GraphPad Software Inc., San Diego, CA, USA). Data are presented as mean ± standard error of mean (SEM). One-way ANOVA was used for comparison among multiple groups, followed by LSD multiple comparison test. The 16S amplicon sequencing analysis was used by the QIIME package data and R programming language. *P* values less than 0.05 were considered statistically significant (*p* < 0.05).

## 3. Results

### 3.1. General conditions of rats

**Figure 1.**
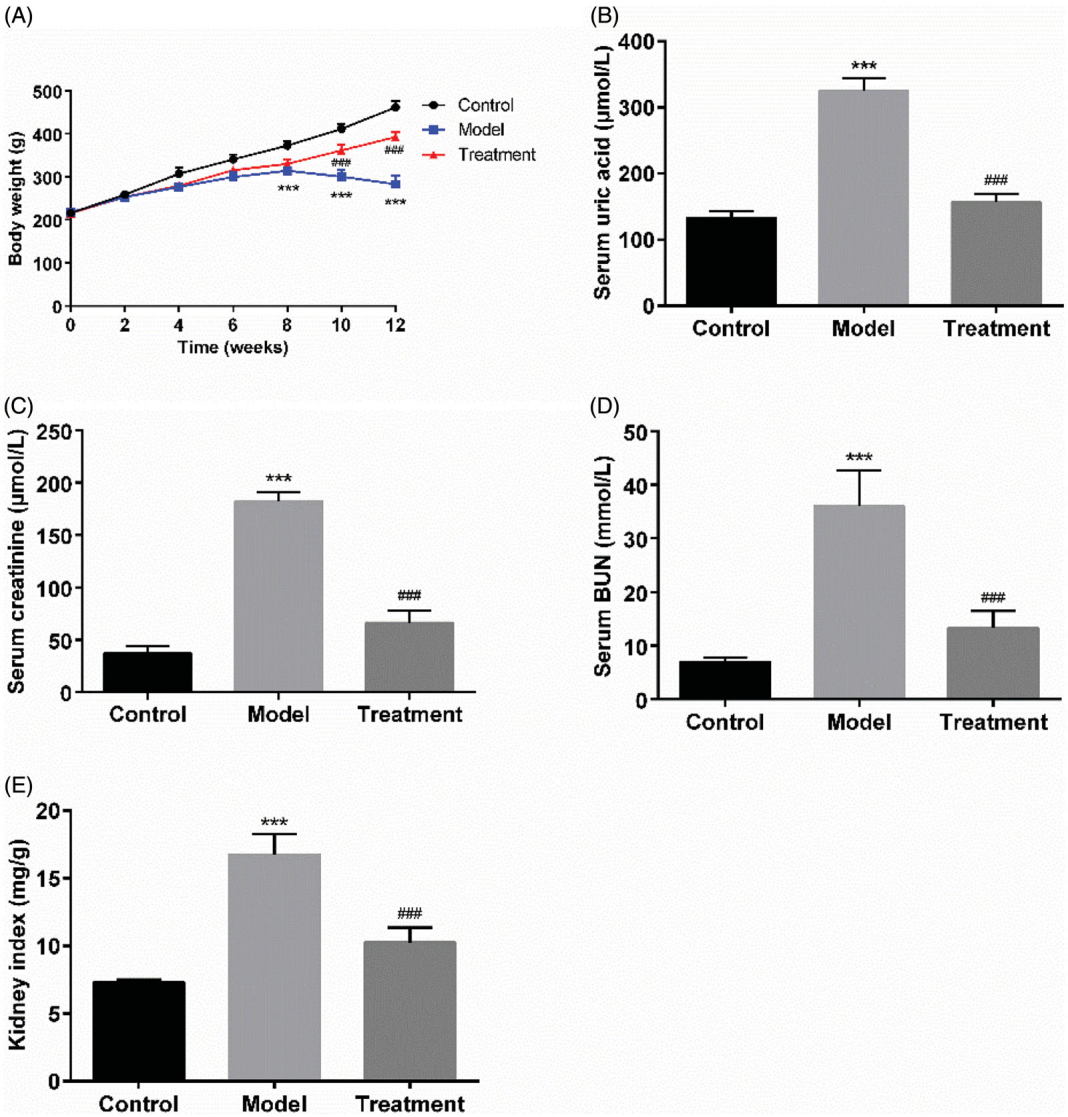
Curcumin improved renal function and reduced serum uric acid of UAN rats. (A) The curves of body weight gain over time. (B) Serum uric acid. (C) Serum creatinine. (D) Serum BUN. (E) Kidney index. Data are presented as mean ± standard error of mean (SEM) in each group (*n* = 10). ***p < 0.001 *vs*. control, *p* < 0.001 *vs*. model.^###^

The rats in the control group were generally in good conditions, with agile movements, smooth fur, normal stool, no obvious changes in food and water intake, urine output. Following model establishment, the rats in the model group showed flagging spirit, decreased activity, loose and dull skin, a poor appetite, and progressive aggravation. Compared with the model group, the symptoms were significantly alleviated in the curcumin treated rats. The results of the body weight growth curve demonstrated that the body mass of rats in the control group showed a natural growth trend and was higher than that in the other groups. The model group exhibited a significant body weight loss on weeks 8, 10, and 12 compared to the control group (*p* < 0.001), while the body weight of the treatment group rats was gradually increased (Figure 1(A)).

### 3.2. Curcumin protected renal function and reduced serum uric acid of UAN rats

The serum level of uric acid in the model group was 325.60 ± 18.65 μmol/L at the end of the experiment, which was increased compared to that of control group (132.80 ± 10.35 μmol/L; *p* < 0.001). Curcumin supplementation significantly reduced the serum uric acid level to 156.80 ± 11.90 μmol/L (*p* < 0.001) compared to the model group (Figure 1(B)). Oral administration of curcumin also reduced the levels of serum creatinine (66.20 ± 11.88 μmol/L of curcumin treatment *vs*.182.20 ± 8.87 μmol/L of UAN model, *p* < 0.001) and BUN (13.33 ± 3.16 mmol/L of curcumin treatment *vs.* 36.04 ± 6.60 mmol/L of UAN model, *p* < 0.001, Figure 1(C,D)). The kidney index in the model group was statistically higher than that in the control group (*p* < 0.001), while the kidney index in the treatment group was closed to that in the control group (Figure 1(E)).

### 3.3. Curcumin alleviated histopathological changes in the kidneys of UAN rats

**Figure 2.**
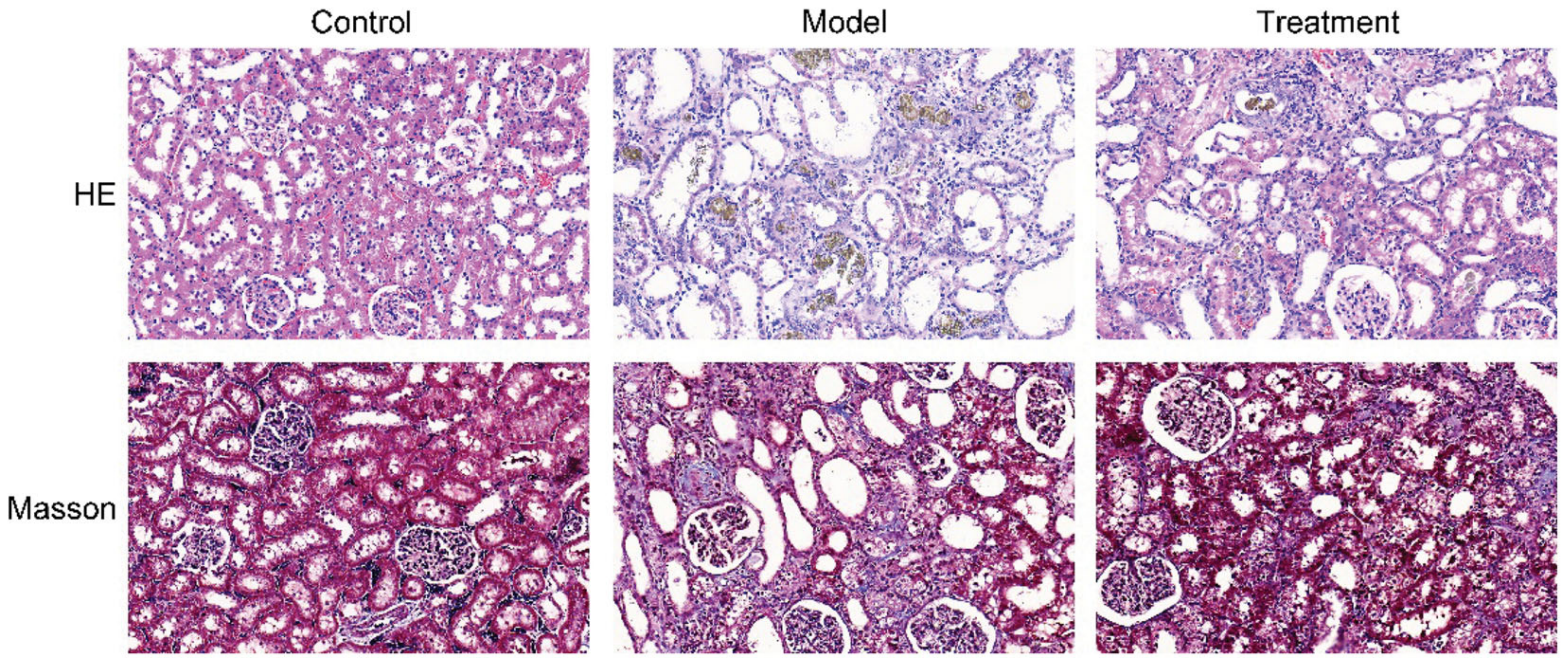
Curcumin alleviated histopathological changes in the kidneys of UAN rats. HE pathological staining and Masson staining. *n* = 10 rats per group. Scale bars = 30 μm.

The results of HE and Masson staining revealed that the kidney tissues of rats in the control group exerted normal morphology without histopathologic changes. HE staining showed that renal pathological changes in the model group was significant, including glomerular sclerosis, mesangial cell proliferation, interstitium edema, matrix widened, inflammatory cell infiltration, and many brown-black uric acid crystals were found in the renal tubules and interstitium, in a needlelike, birefringent radial patterns arrangement. Masson staining results showed that the renal interstitial was broadened and fibrosis was generated in UAN rats. Compared with the model group, the above changes were ameliorated in the treatment group (Figure 2).

### 3.4. Curcumin improved intestinal barrier integrity in UAN rats

**Figure 3.**
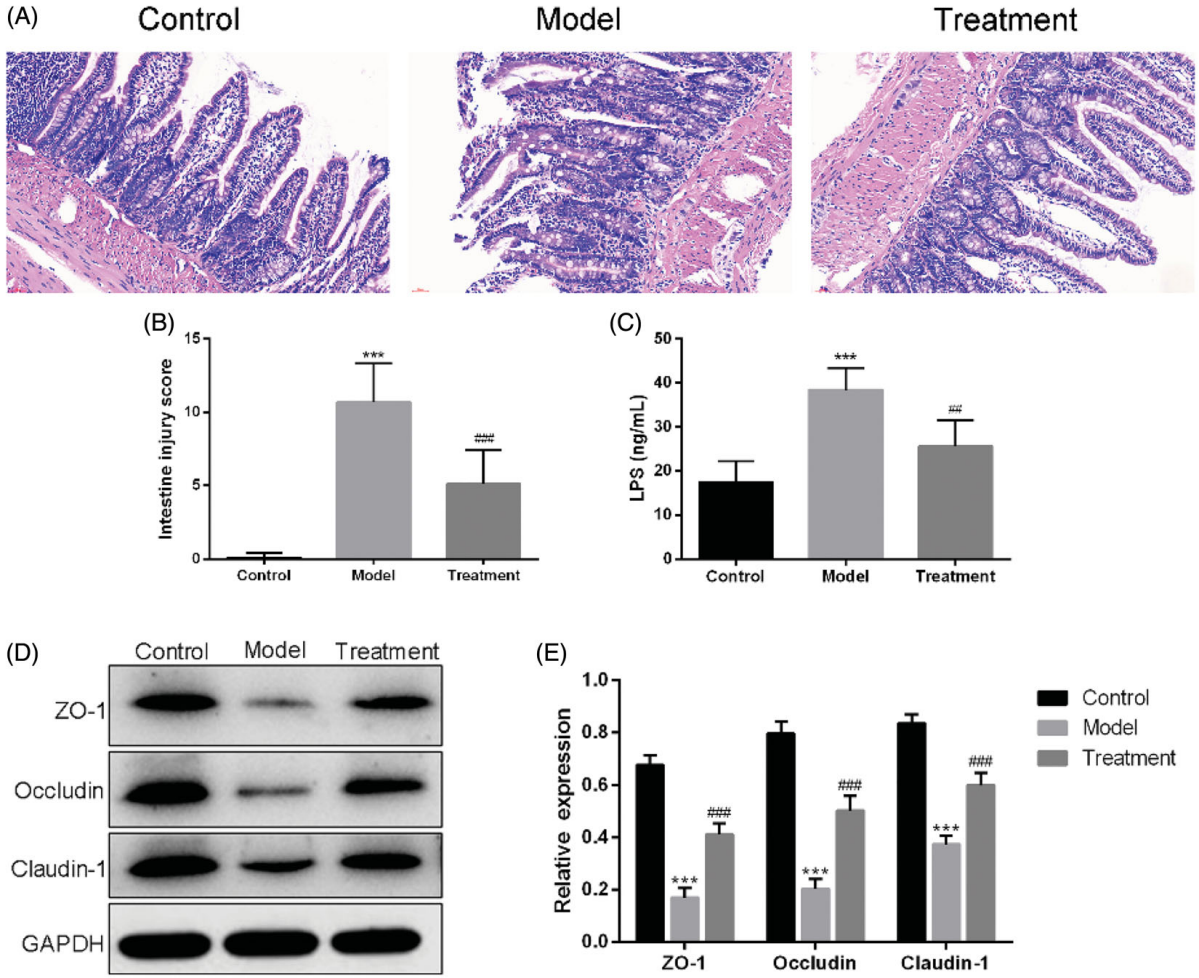
Curcumin improved the intestinal barrier. (A) Images of intestinal tissues of HE staining results. Scale bars = 30 μm. (B) Intestine damage score. (C) Serum LPS. (D) Western blotting results. (E) Relative protein expression levels of ZO-1, occludin and claudin-1 in the ileum of rats. *n* = 10 rats per group. ***p < 0.001 *vs.* control, *p* < 0.01, *p* < 0.001 *vs*. model.^##^^###^

HE staining results showed that, the intestinal tissue structure of rats in the control group was complete, the chorionic glands were arranged regularly without hyperemia or edema, the mucosal layer, submucosa, and lamina propria were intact, and no inflammatory cell infiltration was observed. The intestinal histopathological injuries of rats in the model group were obvious, including villi necrosis, exfoliation or even disappearance, submucosal muscle layer disintegration, lamina propria necrosis and severe inflammatory cell infiltration. The pathological lesions of intestine of UAN rats treated with curcumin were remarkedly improved compared with the model group (Figure 3(A)). The intestine damage score in the model group was significantly higher than that in the control group (*p* < 0.001). After treatment with curcumin, the histological injury score was markedly decreased (Figure 3(B)). In addition, treatment with curcumin obviously decreased plasma LPS concentrations (25.60 ± 5.90 ng/mL of curcumin treatment *vs*. 38.40 ± 4.98 ng/mL of UAN model, *p* < 0.01), as depicted in Figure 3(C).

Western blotting showed that the expression levels of ZO-1, occluding, and claudin-1 were significantly decreased in UAN rats compared with the control group. These expressions were remarkedly higher in the treatment group than in the model group (Figure 3(D,E)).

### 3.5. Microbial diversity analysis

**Figure 4.**
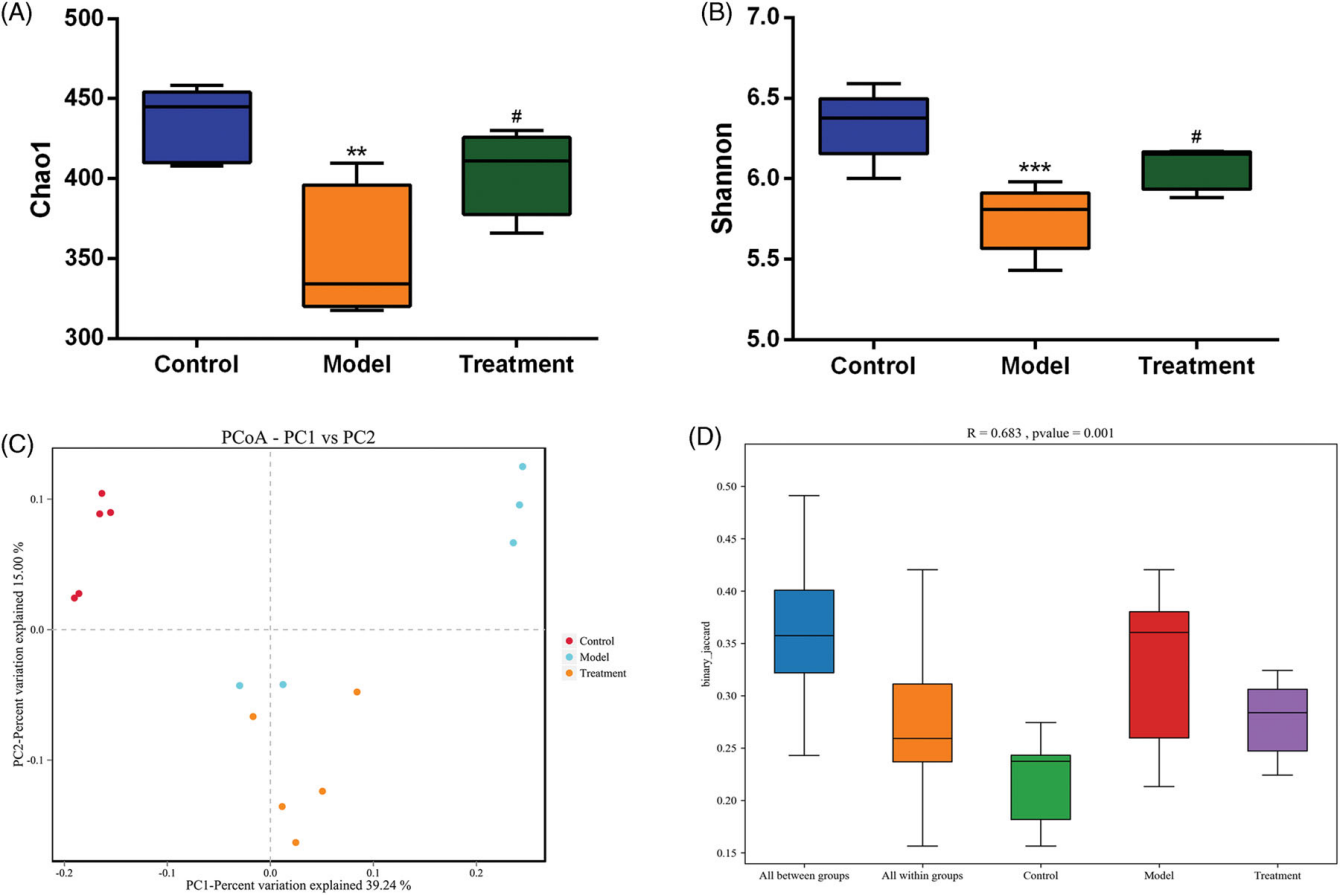
Microbial diversity analysis. (A) Chao1 index. (B) Shannon index. (C) PCoA (Principal coordinates analysis). Dots represent all samples; Different colors represent different groups; X-axis and Y-axis represent two eigenvalues that cause largest difference between samples, they represent the degree of major influence in the form of a percentage. The closer the distance, the higher is the similarity. (D) ANOSIM (analysis of similarities). Y-axis represents Beta distance; The box above 'All between groups' represents the Beta distance data of samples between all groups, while the box above 'All within groups' represents the Beta distance data of samples within all groups. The box below represents the Beta distance data of samples within different groups. The closer the R value (obtained from ANOSIM analysis) is to 1, the higher the difference between groups is than that within groups; the smaller the R value is, the less significant difference is between them; P value less than 0.05 indicates high reliability of the test. *n* = 5 rats per group. **p < 0.01, ***p < 0.001 *vs.* control, *p* < 0.05 *vs*. model.^#^

To reflect the species richness of individual sample and the species diversity, alpha diversity was used to analyze the diversity of microbial communities. Chao1 (Figure 4(A)) and Shannon (Figure 4(B)) diversity indexes were determined using an OTU‐based analysis method. The Chao1 index was used to measure species richness and the Shannon index was used to estimate species diversity. In the case of the same species richness, the higher the evenness of each species in the community is, the higher the community diversity is. Larger Shannon index indicates that the species diversity of the sample is higher [28]. The results indicated that the model group exhibited a loss of α-diversity compared with the control group, while curcumin intervention markedly increased α-diversity in the treatment group compared with the model group.

Beta diversity analysis compares the differences in the composition of the microbial communities between different samples. We applied PCoA based on binary Jaccard algorithm to analyze the beta diversity of the samples (Figure 4(C)). In the rectangular coordinate system, the closer the samples are on the coordinate graph, the higher the similarity is. Analysis of similarity (ANOSIM) was performed to test statistically whether the microbial communities were significantly different among the three experimental groups (Figure 4(D)). The analysis results confirmed that there were significant differences in the community among the three groups and the inter-group distance was significantly greater than the intra-group distance (*R* = 0.683, *p* = 0.001), indicating that the three groups had distinct enterotypes.

### 3.6. Species annotation and taxonomic analysis

**Figure 5.**
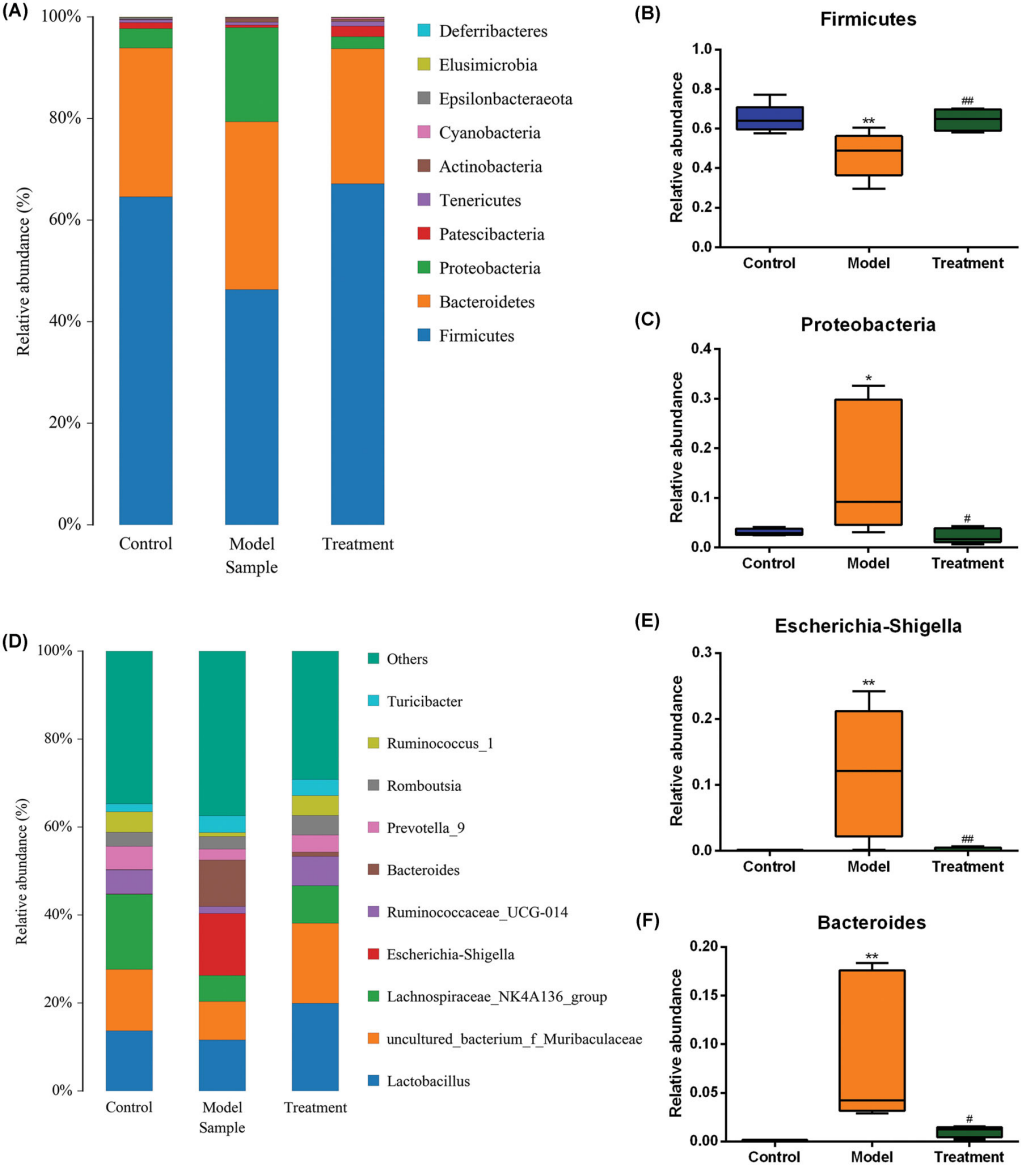
Relative abundance of gut microbiota at phylum (A) and genus (D) levels. One color represents one specie, and the length of the color block presents the relative richness proportion of the species. Abundance of the phyla (B) *Firmicutes*. (C) *Proteobacteria*. Abundance of the genera (E) *Escherichia-Shigella*. (F) *Bacteroides*. *n* = 5 rats per group. *p < 0.05, **p < 0.01 *vs*. control, *p* < 0.05, *p* < 0.01 *vs.* model.^#^^##^

According to the sequencing results, the taxonomics composition distribution histograms of each sample in three groups were shown at phylum and genus levels separately (Figure 5(A,D)). For the best view, only species with top ten richness level were shown, the rest species were combined as others in the chart. The relative compositions of bacterial at phylum and genus levels are presented in Tables 2 and 3. At the phylum level, 10 phyla were detected in all samples, including *Firmicutes*, *Bacteroidetes*, *Proteobacteria*, *Patescibacteria*, *Tenericutes*, *Actinobacteria*, *Cyanobacteria*, *Epsilonbacteraeota*, *Elusimicrobia*, and *Deferribacteres*. As shown in Figure 5(B), the model group had a lower abundance of phylum *Firmicutes* (*p* < 0.01), while curcumin treatment prevented this (*p* < 0.01). Additionally, the abundance of the phylum *Proteobacteria* was expanded by the model group compared with the control (*p* < 0.05), while this increase was restored by the curcumin intervention in the treatment group (Figure 5(C)). At the genus level, we observed that the relative abundance of *Escherichia-Shigella* from family *Enterobacteriaceae* and *Bacteroides* in the model group were significantly increased compared with the control (Figure 5(E,F)). These increases were restored by curcumin intervention.

**Table 2:** The top ten percentages at the phylum level of the three groups (%).

Phylum	Control	Model	Treatment
*Firmicutes*	64.63	46.33	67.17
*Bacteroidetes*	29.28	33.08	26.60
*Proteobacteria*	3.79	18.53	2.34
*Patescibacteria*	1.23	0.51	2.08
*Tenericutes*	0.49	0.55	0.90
*Actinobacteria*	0.13	0.74	0.48
*Cyanobacteria*	0.05	0.14	0.29
*Epsilonbacteraeota*	0.31	0.07	0.08
*Elusimicrobia*	0.06	0.04	0.06
*Deferribacteres*	0.01	0.01	0.00

**Table 3:** The top ten percentages at the genus level of the three groups (%).

Genus	Control	Model	Treatment
*Lactobacillus*	13.69	11.62	19.92
*Uncultured_bacterium_f_Muribaculaceae*	13.93	8.76	18.23
*Lachnospiraceae_NK4A136_group*	17.14	5.87	8.53
*Escherichia-Shigella*	0.01	14.13	0.05
*Ruminococcaceae_UCG-014*	5.45	1.59	6.65
*Bacteroides*	0.14	10.53	0.98
*Prevotella_9*	5.27	2.48	3.86
*Romboutsia*	3.14	2.89	4.46
*Ruminococcus_1*	4.69	0.91	4.50
*Turicibacter*	1.83	3.82	3.73

### 3.7. Significant difference analysis between groups

**Figure 6.**
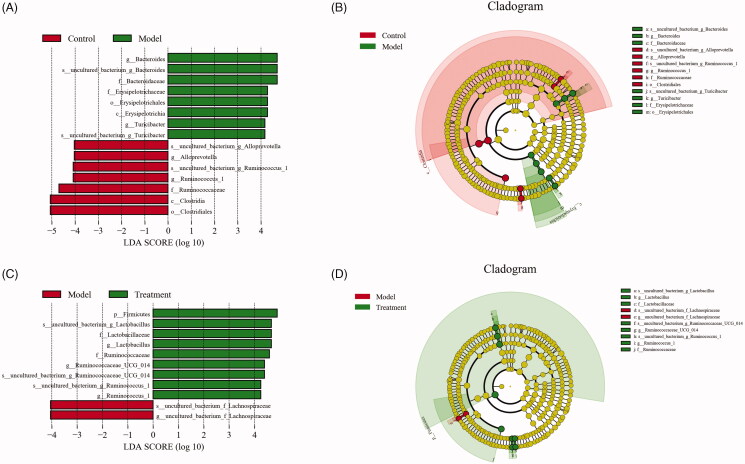
Linear discriminant analysis effect size (LEfSe) and cladograms were applied to identify the microbial taxa with different abundances in three groups. (A, C) The figures show the species whose LDA score is higher than the set value (the default is 4.0). The length of the histogram represents the impact of different species (i.e., LDA Score), different colors represent species in different groups. (B, D) In the cladograms, circles radiating from the inside to the outside represent the different classification ranks from the phylum to the species. Each small circle represents a distinct taxon at the corresponding taxonomic level, with the diameter positively correlated with the relative abundance. The species with no significant difference are colored by yellow and biomarkers are colored based on different groups. Red and green dots represent the core bacterial populations of the respective groups.

The histogram of LDA value distribution and evolutionary branch graph of LEfSe analysis are shown in Figure 6. LEfSe analysis demonstrated higher abundance of the genus *Alloprevotella* belonging to the family of *Prevotellaceae*, two members of family *Ruminococcaceae* and order *Clostridiales* in the control group (Figure 6(A,B)). Compared with the model group, curcumin significantly increased the abundance of the genera *Lactobacillus* and two members from family *Ruminococcaceae* (*Ruminococcus_1* and *Ruminococcaceae_UCG_014*) in the treatment group (Figure 6(C,D)). In addition, we observed that the abundance of *Bacteroides*, *Lachnospiraceae*, and *Turicibacter* of family *Erysipelotrichaceae* were more abundant in the model group.

## 4. Discussion

There is accumulating evidence that hyperuricemia is an independent risk factor for the development and progression of chronic kidney disease [29]. As is well known, sustained high serum concentrations of urate can result in the accumulation of urate crystals in the interstitial of the renal pulp and renal pyramids [30], and stimulates a series of inflammatory reactions to further develop kidney disease [31]. The mechanisms of uric acid-induced renal injury may involve inflammation, endothelial dysfunction, oxidative stress, mitochondrial dysfunction and activation of renin-angiotensin system [32–34]. However, the exact mechanism remains unidentified and there are few safe and effective therapeutic interventions for nephropathy induced by hyperuricemia. In the present study, a uric acid nephropathy rat model was established using adenine and potassium oxonate to investigate renoprotective effects of curcumin. This rodent animal model induced by adenine and potassium oxonate has been reported in the literature [35,36], and had been optimized in our laboratory.

Previous studies have revealed that curcumin alleviates renal interstitial fibrosis and improves renal injury through a variety of mechanisms [37–39], but its impact on the intestinal microflora has not yet been studied. Of note, many studies have demonstrated that the systemic bioavailability of curcumin is extremely low following oral administration, and the accumulation of curcumin in the intestine is high [40–42]. One plausible conjecture is that curcumin intervention may have potential effects on gut flora with the majority of the ingested curcumin exposed to intestine, which in turn exerts systemic effects. As shown above, curcumin markedly altered the gut microbiota diversity and the community compositional structure of the intestinal microbiota. In this study, the abundance and diversity of the intestinal flora of the model group rats were significantly reduced compared with that in the control group, while curcumin intervention exhibited a significant callback effect on bacterial diversity. Combined with the multiple outlier samples of rats in the model group in the PCoA analysis, it indicated that there was an imbalance in intestinal flora during the progression of UAN, and curcumin could improve this disorder to some extent.

Based on the theory of the gut-kidney axis, we dug deeper into the alteration of intestinal microflora composition that could induce kidney damage. Under normal circumstances, human gut microbiome is in a state of equilibrium, with the dominating bacterial phyla being *Firmicutes* and *Bacteroidetes* [43]. The dominant bacterial phyla of each group of rats in the experiment were similar to that of human, and the three dominant bacteria, *Firmicutes*, *Bacteroidetes*, and *Proteobacteria*, accounted for more than 90% of the total intestinal flora. Compared with the control group, the abundance of *Firmicutes* phylum in the model group was decreased, while the abundance of *Proteobacteria* phylum was increased. This result was in accordance with previous research findings [44]. *Proteobacteria* is a major phylum of Gram-negative bacteria which includes a wide variety of opportunistic pathogens, such as those of the genera *Escherichia*, *Vibrio*, and *Salmonella*. At the genus level, we found that amounts of *Escherichia-Shigella* and *Bacteroides* were significantly increased in the model group. In 5/6 nephrectomized rats, Kikuchi *et al.* [45] found that serum uremic toxins levels were positively correlated with the relative abundance of *Bacteroides* genus. These species have genes potentially encoding for necessary enzymes for tryptophan and tyrosine synthesis, which indicates that they play an important role in the process of uremic toxins production. *Escherichia-Shigella* in genus level, a member of the family *Enterobacteriaceae*, was a Gram-negative bacillus and opportunistic pathogen, containing urease, indole-, and p-cresol-forming enzymes. Bacterial urease metabolizes urea to ammonia and carbon dioxide. In chronic kidney disease, the accumulation of uremic toxins in the circulation leads to a large influx of urea into the gastrointestinal tract, in which urea is hydrolyzed to ammonia then to ammonium hydroxide by urease-possessing microbiota, resulting in increased intestinal lumen pH and a local inflammatory response [46]. Notably, excessive fermentation of dietary protein and amino acids by intestinal flora results in the production of toxic metabolites, such as phenols and indoles [47]. For instance, Studies have demonstrated that dietary tryptophan is metabolized to indole by *Escherichia coli* in the intestine, which is further metabolized to indoxyl sulfate (IS) by the liver [48]. Similarly, p-cresol is a phenolic compound produced from tyrosine and phenylalanine through the intestinal bacterium fermentation and further metabolized to p-cresol sulfate (PCS) in the liver [49]. Hence, imbalance in these two metabolites levels might indicate that the intestinal microflora was disrupted under the condition of nephropathy induced by hyperuricemia. Meanwhile, as protein-bound uremic toxins, serum PCS and IS levels were closely associated with progression of renal dysfunction and all-cause mortality [50–52]. Moreover, results from a recent work suggested that high *Escherichia-Shigella* abundance appeared to be closely related to abnormal intestinal flora composition (i.e., dysbiosis) [53]. All the evidences above implied that *Escherichia-Shigella* may be an essential biomarker for the occurrence and development of kidney disease.

In the present study, LEfSe analysis based on the OTU level was used to find the biomarkers between groups. The results demonstrated that *Alloprevotella* belonging to the family of *Prevotellaceae* and *Ruminococcaceae* from the order *Clostridiales* were the dominant bacteria in the control group, which were butyrate-producing microbes and benefited the host by metabolizing plant cell wall dietary fiber into short-chain fatty acids (SCFAs) [54–56]. SCFAs supplementation can significantly improve intestinal barrier function and renal function [57,58]. In the model group, the abundances of *Erysipelotrichaceae* family, *Bacteroides*, *Lachnospiraceae*, and *Turicibacter* genera were significantly increased, indicating that these bacteria might play important roles in the progression of UAN. Microbes from the family *Erysipelotrichaceae* were closely associated with intestinal inflammation [59]. Additionally, in a recent study, *Erysipelotrichaceae* showed positive correlation with both BUN in plasma and kidney index [60], which implied that the strain might be detrimental to renal function. Disturbances of the normal gut microbiome in chronic kidney disease showed that *Escherichia-Shigella*, *Bacteroides*, *Turicibacter*, *Blautia*, and *Lachnospiraceae* were enriched, which were similar to our findings. These strains are related with systemic inflammation, intestinal barrier integrity and renal fibrosis [61–63]. After curcumin intervention, *Lactobacillus* became the dominant bacteria in the treatment group. As the most common probiotic genus, increased abundance of *Lactobacillus* can increase the production of SCFAs, improve barrier function, minimize uremic toxin retention in body, reduce chronic intestinal and systemic inflammations, and then protect renal function [64–66]. The analysis described above suggests that curcumin intervention in UAN rats can decrease pathogenic bacteria which possess urease, indole-, and p-cresol-forming enzymes, such as *Escherichia-Shigella*, and increase beneficial bacteria which possess SCFAs forming enzymes, such as *Lactobacillus*.

Apart from modulating the gut microbiota composition, curcumin treatment also maintained intestinal barrier integrity and lowered the circulating levels of LPS. The intestinal mucosal barrier is composed of mechanical, chemical, immunological, and biological barriers [67]. The mechanical barrier composed of tight junctions between epithelial cells is the most important part of intestinal mucosal barrier, and can prevent the translocation of bacteria and microbial products to the blood under normal circumstances [12]. ZO-1, occluding, and claudin-1 are important tightly linked proteins contributing to barrier function, and decreased expression levels of these proteins may lead to increased permeability of the intestinal mucosal barrier. In our study, the expressions of these tight junction proteins were significantly decreased in the model group, indicating that the intestinal barrier had been damaged. We speculate that this may be associated with the intestinal flora disturbance which can produce massive intestine-derived uremic toxins, leading to intestinal mucosal edema, atrophy, and inflammation. At the same time, intestinal pathogenic bacteria can induce the disruption of structural barriers by changing intestinal tight junction proteins [68]. The intestinal barrier dysfunction increases intestinal permeability and results in the translocation of conditional pathogenic bacteria and LPS into the blood circulation, which causes severe metabolic endotoxemia and systemic inflammation, and in turn exacerbates renal damage [69]. According to this, curcumin treatment may prevent intestinal bacterial translocation, and reduce the inflammatory response by modifying the intestinal microflora and fortifying the intestinal barrier, thus indirectly protecting the renal function in UAN rats.

Meanwhile, further researches are required to determine whether the modulating action of curcumin on intestinal flora in the context of UAN is dose- and time-dependent. In the future, our group will conduct in-depth studies to address these issues and expect to provide a novel avenue for the prevention and treatment of uric acid nephropathy.

## 5. Conclusions

In conclusion, this research demonstrated that curcumin lowers the level of uremic toxins, ameliorates inflammation and fibrosis in the kidneys by regulating the structure of intestinal flora and improving intestinal permeability, which may be related to increase in beneficial bacteria (e.g., *Lactobacillus* and *Ruminococcaceae*) and decrease in pathogenic bacteria (e.g., *Bacteroides*, *Lachnospiraceae*, and *Escherichia-Shigella*). Our findings revealed a new role of curcumin in renal protection. However, the concrete molecular mechanism of curcumin in treating uric acid nephropathy needs to be further investigated.

## Ethical approval

The study was conducted according to the guidelines of the Declaration of Helsinki, and approved by the Ethics Committee of Animal Experiments of the Affiliated Hospital of Qingdao University (QYFYWZLL26159; 2020.09.01).
